# Draft Genome Sequence of the Cellulolytic Strain *Clostridium* sp. Bc-iso-3 Isolated from an Industrial-Scale Anaerobic Digester

**DOI:** 10.1128/genomeA.01188-16

**Published:** 2016-10-27

**Authors:** Li Sun, Anna Schnürer

**Affiliations:** Department of Microbiology, Swedish University of Agricultural Science, Uppsala BioCenter, Uppsala, Sweden

## Abstract

*Clostridium* sp. Bc-iso-3 is a cellulolytic strain isolated from a Swedish industrial-scale biogas digester. Here, we present the draft genome sequence of this strain, which consists of four contigs with a total length of 4,327,139 bp and an average coverage of 312.97×.

## GENOME ANNOUNCEMENT

Cellulosic materials represent the most abundant biopolymer on earth (1), and microbial cellulose utilization is a key step in the global carbon cycle (2). Consequently, cellulases produced by microorganisms are not only of importance in nature but are also important for various applications in industrial processes (3). Anaerobic digestion represents one such interesting application, and during this process organic materials can be converted to renewable energy in the form of biogas. An array of different microbial steps is required for this process, and the first step, the hydrolysis, represents a key step during the degradation of plant-based materials (4). With the aim to further understand the hydrolysis of lignocellulose in biogas digester isolations were performed. A cellulolytic strain, *Clostridium* sp. Bc-iso-3, was retrieved from an industrial anaerobic digester (CSTR) in Sweden. Based on a full-length 16S rRNA gene analysis, its closest relative is *Clostridium thermocellum* DSM 2360 with 96% sequence identity. At the time of sampling, this digester was operated at 38°C using source-separated municipal organic waste and slaughterhouse waste as main substrates. The isolation was initiated by an enrichment using cellulose as a substrate, followed by a procedure described previously (5) using cellulose (5 g/L) as the energy and carbon source. The isolation was performed at the Department of Microbiology, Swedish University of Agricultural Sciences. The genomic DNA was isolated with the DNeasy blood and tissue kit (Qiagen, Inc., Valencia, CA, USA), followed by a cleanup step using the PowerClean Pro DNA cleanup kit (MO BIO Laboratories, Carlsbad, CA, USA). The genomic DNA was then used for constructing a 10-kb SMRTbell library and sequenced using the PacBio RSII single-molecule real-time (SMRT) sequencing platform; the raw reads were assembled with HGAP version 2.0 (Pacific Biosciences, Menlo Park, CA, USA) (6). In total, 137,571 post-filter reads were obtained from two SMRT cells with a mean read length of 11,675 bp. The draft genome of *Clostridium* sp. Bc-iso-3 consists of four contigs with a total length of 4,327,139 bp and an average coverage of 312.97×, among which the max contig length is 4,269,335 bp with a coverage of 315.87×.

The draft genome has a G+C content of 38.2%. In total, four sets of 5S-23S-16S rRNA genes were predicted using RNAmmer (7), and 56 tRNA genes covering all 20 amino acids were predicted using tRNAscan-SE (8). Using PRODIGAL (Prokaryotic Dynamic Programming Gene-finding Algorithm, version 2.6.3), a total of 3,711 coding sequences were predicted (9).

The carbohydrate metabolism enzymes (CAZyme) were annotated using dbCAN (10). In total, 81 CAZymes were found, including one auxiliary activity, 23 carbohydrate-binding modules, nine carbohydrate esterases, 29 glycoside hydrolases, 16 glycosyltransferases, and three polysaccharide lyases.

Accessibility of this genome sequence will further allow a detailed analysis of anaerobic cellulose degradation pathways, as well as a comparative analysis with other bacteria having the ability to degrade cellulose.

### Accession number(s).

This whole-genome shotgun project has been deposited at DDBJ/ENA/GenBank under the accession number LXSB00000000. The version described in this paper is the first version, LXSB01000000.
